# Temporal Discrimination and Proprioceptive Impairment in Parkinson’s Disease: Evidence for Distinct Sensory Dysfunction Mechanisms

**DOI:** 10.1155/padi/8849526

**Published:** 2026-02-03

**Authors:** Annika Junge, Jens Volkmann, Daniel Zeller, Thorsten M. Odorfer

**Affiliations:** ^1^ Department of Neurology, University Hospital Würzburg, Würzburg, 97080, Bavaria, Germany, ukw.de

## Abstract

**Background:**

Parkinson’s disease (PD) is characterized by motor symptoms but also includes nonmotor impairments such as sensory disturbances. Temporal discrimination (TD) deficits have been repeatedly demonstrated, while proprioceptive dysfunction is also common in PD. The exact significance of these alterations, and whether they represent related aspects of a common pathophysiological process, remains elusive.

**Methods:**

We investigated somatosensory and kinesthetic TD as well as proprioceptive accuracy in 20 PD patients and compared 20 age‐ and sex‐matched healthy controls (HCs). Somatosensory TD threshold (STDT) was assessed using paired cutaneous electrical stimuli and TD motor thresholds (TDMTs) by electrically induced wrist flexions. Proprioception was measured with two tasks requiring wrist flexion to predefined angles (LED task) or to reproduce angular ranges (ARROW task), without visual feedback. Clinical assessment comprised MDS‐UPDRS III, Hoehn and Yahr stage, levodopa dosage, and disease duration.

**Results:**

PD patients exhibited significantly elevated thresholds compared to HC for both STDT (120.3 ± 42.3 ms vs. 80.8 ± 17.1 ms, *p* = 0.001) and TDMT (107.1 ± 43.7 ms vs. 77.0 ± 16.3 ms, *p* = 0.011). Proprioceptive errors were also higher in PD for the LED (6.7° ± 2.2° vs. 3.2° ± 1.9°, *p* < 0.001) and ARROW tasks (14.2° ± 3.4° vs. 2.1° ± 0.8°,*p* < 0.001). No significant correlations were observed between TD, proprioceptive measures, or clinical severity indices.

**Conclusions:**

PD patients show pronounced impairments in both TD and proprioceptive accuracy, confirming sensory processing deficits beyond motor dysfunction. The absence of correlations suggests distinct mechanisms, highlighting the need for further neurophysiological research.

## 1. Introduction

Parkinson’s disease (PD) is clinically defined by the presence of at least two of its cardinal motor symptoms: bradykinesia, rigidity, and resting tremor [1]. Nevertheless, patients almost invariably experience a wide range of nonmotor symptoms, including psychiatric disturbances, autonomic dysfunctions, sleep impairments, and sensory abnormalities (for a review, see [2]). While sensory disturbances most commonly manifest as olfactory and visual impairments or pain [2], recent years have witnessed increasing evidence of subclinical alterations in somatosensory processing, encompassing tactile, nociceptive, thermal, and proprioceptive domains (for a review, see [3]).

Within this context, temporal discrimination (TD), the ability to perceive two sensory stimuli presented in close temporal succession as distinct events, has also been investigated [4]. Numerous studies have shown that individuals with PD exhibit elevated TD thresholds compared to healthy controls (HCs), suggesting a diminished temporal resolution of sensory processing [5–7]. However, the precise significance of these findings, particularly with respect to their potential contribution to the pathophysiological mechanisms of PD, remains unclear [3]. Neuroanatomical considerations point toward a possible disruption of interactions between basal ganglia structures and the supplementary motor area (SMA) as an underlying factor [3, 8, 9].

Our previous research suggested an association between alterations in TD and proprioceptive deficits, at least in healthy individuals and in patients with polyneuropathy [10]. Given that proprioceptive impairments are also highly prevalent in PD (for a review, see [11]), we considered a systematic investigation of TD in both somatosensory and kinesthetic domains, combined with a straightforward proprioceptive task, to be a promising approach. Rather than assuming a direct mechanistic linkage between these domains, we adopted an exploratory approach to determine (i) whether both TD and proprioceptive accuracy are impaired in PD and (ii) whether potential associations between these measures can be detected. Such a combined assessment may help clarify whether distinct or partially overlapping mechanisms contribute to different aspects of sensory dysfunction in PD.

## 2. Materials and Methods

The protocol adhered to the principles of the Declaration of Helsinki and was approved by the Ethics Committee at the Medical Faculty of the University of Wuerzburg (reference number: 17/20‐sc). Written informed consent to participate in the study was obtained from all participants.

### 2.1. Participants

We recruited 20 patients with PD at our clinic and the outpatient clinic for movement disorders. The clinical diagnosis was made by a physician with specialist experience in movement disorders. The clinical examination comprised the Part III (motor scale) of the Movement Disorders Society Unified PD Rating Scale (MDS‐UPDRS) [12] and the determination of the current Hoehn and Yahr stage [13]. The demographic data and precise clinical characterization are documented in Table 1. The study investigations were conducted in a clinical ON state condition, as rated concordantly by the patient and the investigator. In addition to oral medical treatment, pump therapy and deep brain stimulation (DBS) therapy were permitted. Further details of the therapy can be found in Table 1. The HC group consisted of 20 age‐ and sex‐matched individuals previously recruited for an earlier study using identical experimental procedures, equipment, and examiner training [10]. All assessments were conducted in the same laboratory environment and under supervision of the same investigator. Data acquisition in the HC cohort preceded patient data collection by approximately 5 years. To ensure methodological comparability, all measurements for the present analysis were reevaluated under identical preprocessing and scoring criteria. All subjects underwent assessment for clinical deficits in deep sensation and proprioception, with testing of both pallesthesia and the positional sense of the hallux. Individuals with abnormalities were excluded, since peripheral afferent dysfunction could interfere with the TD assessment.

**TABLE 1:** Demographic and clinical characteristics of patients with Parkinson′s disease.

No.	Sex	Age	AoO	H&Y	UPDRS III	PD type	DBS	Daily LEDD	Other CNS acting medication^∗^
1	m	64	51	2	24	AR	yes	828.5	
2	m	58	35	3	24	AR	yes	250	
3	m	81	73	4	67	AR	no	1050	Gabapentin
4	m	61	47	3	36	AR	yes	919	
5	f	68	60	2	12	AR	no	984.3	
6	m	66	57	2‐3	64	ART	no	1263.8	Quetiapine
7	m	70	63	2	28	T	yes	150	
8	m	60	47	2	35	AR	no	936.8	
9	f	64	61	1	9	ART	no	149.3	
10	m	81	71	3	22	AR	no	1395	Mirtazapine
11	f	59	57	1	17	ART	no	399.3	Primidone
12	f	55	50	2‐3	19	AR	yes	532	
13	f	54	40	3	10	AR	yes	97.5	Pregabalin
14	m	68	58	2	14	AR	yes	300	Mirtazapine
15	m	61	58	1	15	AR	no	225	Clonazepam
16	m	66	58	2	32	ART	no	648.75	Gabapentin
17	f	69	63	3	53	AR	no	4147.2	
18	m	69	62	2	19	ART	no	756.3	
19	f	59	49	3	26	ART	no	771	
20	m	53	41	3	11	AR	yes	232.5	

*Note:* H&Y = Hoehn and Yahr stage; LEDD = levodopa equivalent dose in mg; AR = akinetic‐rigid type; T = tremor‐dominant type; ART = equivalent type.

Abbreviations: AoA = age of onset; CNS = central nervous system; DBS = deep brain stimulation; PD = Parkinson’s disease; UPDRS III = Unified Parkinson’s Disease Rating Scale Part III.

^∗^Concomitant to PD medication.

### 2.2. Measures of TD

This study investigated two types of TD: somatosensory TD and kinesthetic TD. The former assesses the perception of superficial stimuli on the skin, while the latter involves the perception of electrically induced muscle movements.

To measure somatosensory TD threshold (STDT), surface electrodes (anode and cathode, each 1 mm in diameter and spaced 1.5 cm apart) were placed over the thenar region. Electrical stimulation was delivered using a constant current stimulator (Digitimer, Welwyn Garden City, United Kingdom), applying paired square‐wave pulses with a duration of 0.2 ms. Stimulation intensity was individualized by gradually increasing the current until participants could reliably perceive the stimuli in 10 out of 10 trials. Stimuli were presented in both ascending and descending sequences, starting at either 30 or 300 ms, with 5 ms increments. The STDT was defined as the shortest interstimulus interval (ISI) at which participants perceived two distinct pulses. The average of four trials was used for further analysis.

To assess kinesthetic TD, the TD motor threshold (TDMT) was determined. For this purpose, a monopolar EMG needle electrode was inserted into the right flexor carpi radialis muscle, and the anode was a surface electrode placed 3‐4 cm distal to the cathode. Using the same stimulation parameters as described above, the TDMT was defined as the shortest ISI at which participants could clearly distinguish two separate muscle contractions (i.e., wrist flexions) in three consecutive trials. Again, the mean of four attempts was used for analysis.

### 2.3. Proprioception Task

Proprioception of hand movements was assessed as published before [10] by using custom‐made goniometers, with visual feedback excluded. Wrist flexion was measured within a range of 0°–75°, where 0° marked the starting position, corresponding to 15° of wrist extension according to the neutral‐zero method. The goniometer enabled the investigator to monitor and quantify movements on an external scale hidden from the participant. To isolate wrist flexion, the hand was stabilized with splints, preventing other joint movements. Participants performed two proprioceptive tasks, initiating movement only upon the investigator’s cue and aiming for high precision.

LED task (Figure 1(a)): Participants pointed to illuminated LEDs placed at 15°, 30°, 45°, 60°, or 75° by executing a single, quick movement. Each LED was presented three times in random order. The examiner recorded the reached position, and participants returned to the start before the next trial.

FIGURE 1Custom‐made goniometers to quantify pointing errors of hand movements. (a) LED task: wrist flexion toward an LED light without visual feedback. (b) ARROW task: example of a computer screen instruction (left part) and the corresponding active movement of the upper limb.

**Figure:**
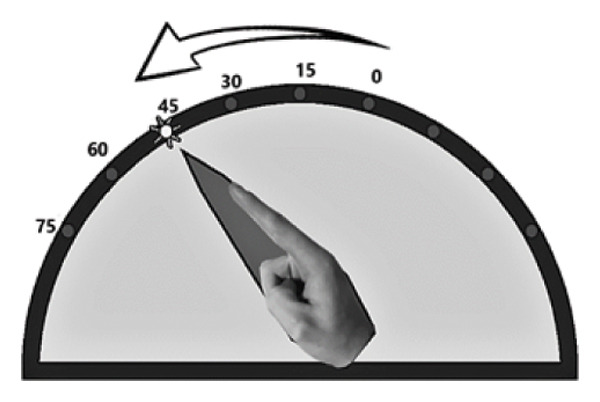
(a)

**Figure:**
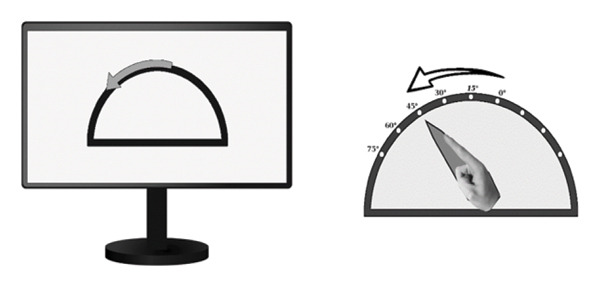
(b)

ARROW task (Figure 1(b)): Participants matched the angular extent of curved arrows (15°, 30°, or 45°) displayed on a screen, imitating the indicated range of movement. Each arrow was shown three times in randomized order.

Pointing errors in degrees were used as a measure of proprioceptive accuracy.

### 2.4. Statistics

SPSS software (IBM, Armonk, NY, USA) was used for statistical analyses. According to the Shapiro‐–Wilk test, data were not normally distributed. Therefore, the Mann–Whitney *U* test was used for group comparisons and the Spearman test for correlations. Statistical significance was set at *p* < 0.05. Correlation analyses were considered exploratory. Given the sample size and the number of correlation tests performed, no formal correction for multiple comparisons (e.g., false discovery rate) was applied. As no correlations reached statistical significance, the primary concern relates to limited statistical power and the risk of false‐negative findings.

## 3. Results

Twenty PD patients and 20 controls (HC) were included, with 7 women and 13 men in the PD group (vs. 9 women and 11 men in the HC group). The mean age was 64.3 ± 7.7 years in the PD group and 63.9 ± 8.5 years in the HC group (*p* = 0.862). Detailed information on the patient group is summarized in Table 1. The median MDS‐UPDRS Part III score was 23 [range: 9–67] points. All Hoehn and Yahr stages were represented, with the majority of patients being in Stages 2 and 3. The average disease duration was 9.3 ± 4.8 years. Seven patients presented with a clinically relevant tremor syndrome PD. Mean levodopa equivalent daily dose (LEDD) (calculated as suggested by Nyholm and Jost [14]) was 801.8 ± 879.8 mg. Seven patients were treated with DBS, 1 patient received foslevodopa subcutaneously.

Regarding TD paradigms, the threshold levels of the PD patients were significantly higher than those of the HC group: STDT PD group 120.3 ± 42.3 ms versus STDT HC group 80.8 ± 17.1 ms (*p* = 0.001; Figure 2(a)) and TDMT PD group 107.1 ± 43.7 ms versus TDMT HC group 77.0 ± 16.3 ms (*p* = 0.011; Figure 2(b)).

FIGURE 2Somatosensory temporal discrimination threshold (STDT; (a)) and temporal discrimination motor threshold (TDMT; (b)) in Parkinson’s disease (PD) versus healthy controls (HC). The asterisk indicates *p* < 0.05.

**Figure:**
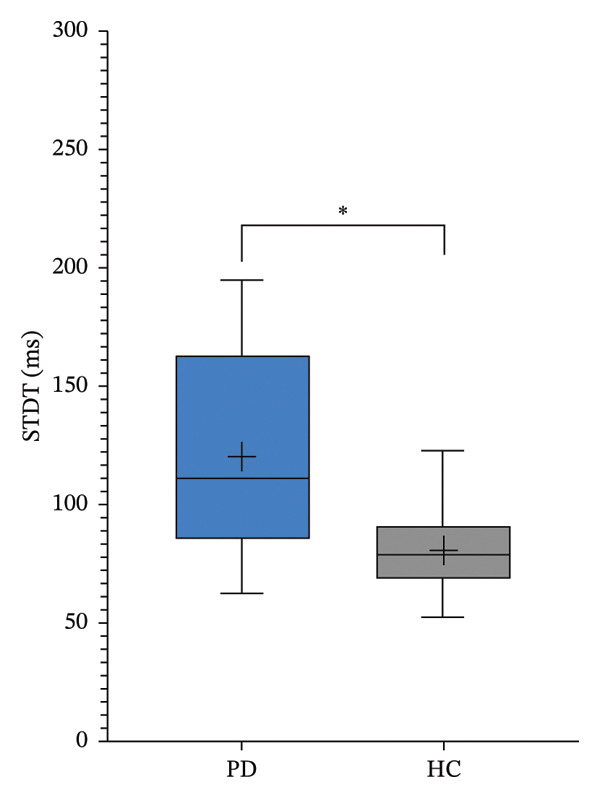
(a)

**Figure:**
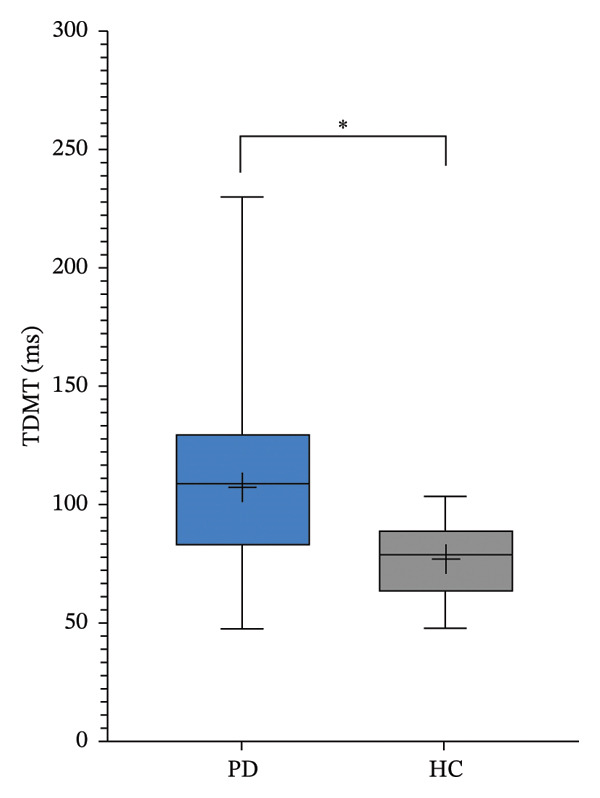
(b)

In both proprioception tests, patients with PD showed significantly higher error rates than HCs (LED: 6.7° ± 2.2° vs. 3.2° ± 1.9°, *p* < 0.001; ARROW: 14.2° ± 3.4° vs. 2.1° ± 0.8°, *p* < 0.001; Figures 3(a) and 3(b)).

FIGURE 3Pointing errors in LED (a) and ARROW (b) task in Parkinson’s disease (PD) versus healthy controls (HC). The asterisk indicates *p* < 0.05.

**Figure:**
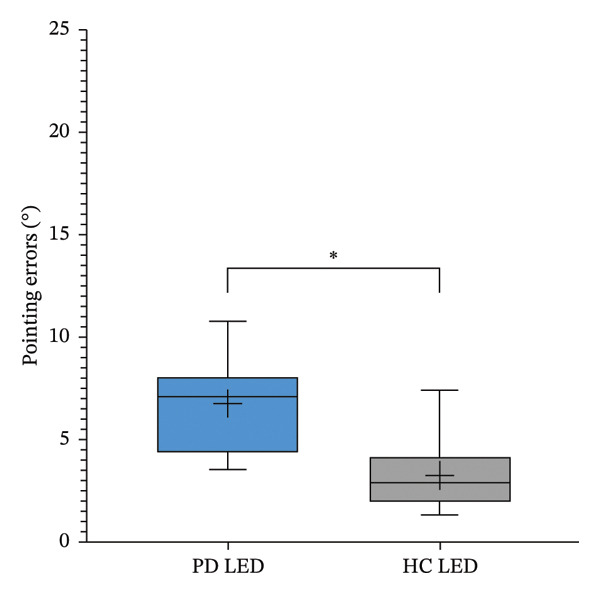
(a)

**Figure:**
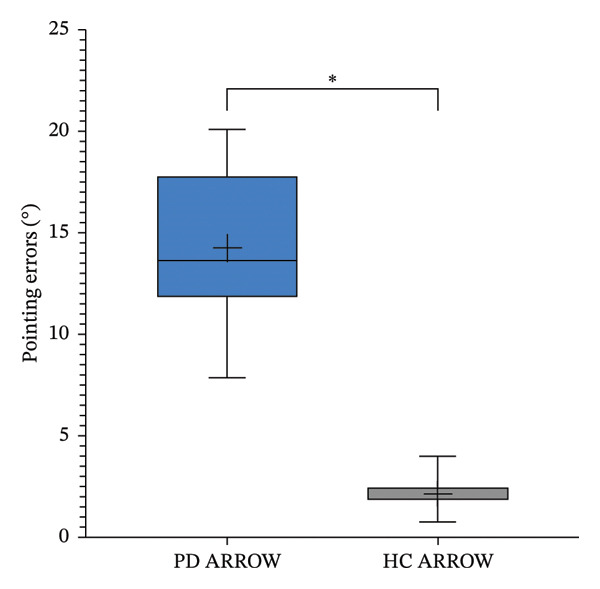
(b)

No significant results could be obtained when examining correlations between TD and behavioral measurements and clinical characteristics (MDS‐UPDRS motor score, Hoehn and Yahr stage, LEDD, and disease duration). In addition, no group differences were observed for TD and the pointing tasks when PD patients were divided into subgroups (tremor vs. no tremor; DBS vs. no DBS).

## 4. Discussion

### 4.1. Summary of Findings

The present study demonstrates that individuals with PD, examined in a naturalistic clinical cohort, exhibit significant impairments in both TD thresholds (STDT and TDMT) and proprioceptive accuracy compared to HCs. Regarding hypothesis (i), these results confirm that both TD and proprioceptive accuracy are compromised in PD. However, no correlations were observed between TD measures and pointing errors, nor between sensory deficits and clinical severity indices (e.g., UPDRS motor score, Hoehn and Yahr stage, disease duration, or levodopa dose). With respect to hypothesis (ii), the absence of significant associations indicates that TD abnormalities and proprioceptive deficits do not necessarily reflect a single, unified sensory impairment. Given the exploratory nature of the correlation analyses and limited statistical power, this absence of significant associations should be interpreted as no evidence of association in this sample rather than evidence for distinct underlying mechanisms. Consequently, the hypotheses of a direct linkage between TD and proprioceptive dysfunction were only partially supported.

### 4.2. Integration With Existing Literature

Our findings corroborate previous reports of elevated STDT in PD [5–7], confirming the reliability of this observation. In contrast to some studies that suggested a link between STDT alterations and clinical severity [5, 7], we found no association between the magnitude of TD deficits and parkinsonian phenotype, in line with other studies that likewise failed to demonstrate such a relationship [15, 16]. A consistent correlation between phenotype and endophenotype would have provided strong evidence for the pathophysiological relevance of these measurable changes, but current evidence remains inconclusive. Similarly, potential modulators such as disease duration [17] and levodopa treatment [5, 7, 18, 19] were not evident in our dataset. Notably, our assessment differed from previous studies: all patients were evaluated in the clinically defined “ON” state, correlating daily levodopa dose with TD levels, whereas earlier work often included both “ON” and “OFF” states. [5, 7, 18, 19] The present findings therefore reflect sensory processing in treated PD during the ON condition and cannot disentangle disease‐related effects from dopaminergic or medication‐related influences. With respect to DBS, evidence remains limited, as one study has reported a worsening of STDT performance in the stimulation “ON” condition [15]. Regarding motor tasks, prior studies relating STDT to performance yielded inconsistent results, with some showing correlations with wrist flexion velocity [5] or coin rotation [20, 21], while finding variable outcomes for finger tapping [5, 20, 21].

Compared to STDT, TDMT has been less extensively studied. Preliminary evidence indicates elevated TDMT in PD [16] and in other movement disorders such as writer’s cramp [22], essential tremor [23], and cervical dystonia [24]. To our knowledge, movement tasks primarily demanding proprioception have not previously been evaluated using a combined STDT and TDMT approach.

### 4.3. Proprioceptive Dysfunction in PD

Proprioceptive deficits in PD have been extensively documented (for a review, see [3]), including impairments in passive movement estimation [25, 26], arm proprioception [27, 28], and reaching with visual feedback [29]. The influence of dopaminergic medication remains debated, with studies reporting improvements [30], deteriorations [27, 29], or no effect [25, 31]. DBS appears to ameliorate proprioceptive dysfunction [28], suggesting that altered subthalamic nucleus activity may modulate proprioceptive processing in the somatosensory cortex [3]. Despite extensive investigation, the precise nature and clinical significance of proprioceptive deficits in PD remain unclear due to methodological heterogeneity and multiple contributing factors.

### 4.4. Interpretation of Findings

To interpret the present findings, it is important to consider current models of temporal somatosensory dysfunction and its relationship to motor control (for a review, see [3, 32]). Lee et al. argue that abnormal temporal processing disrupts the sensorimotor feedback loop, thereby contributing to motor dysfunction [32]. Conte et al. expand this framework by distinguishing between perceptual deficits (e.g., increased sensory thresholds) and impaired sensorimotor integration, where the translation of sensory input into motor output becomes dysfunctional. According to their model, reduced dopaminergic modulation, impaired signal‐to‐noise discrimination, and decreased response specificity all contribute to abnormal sensory processing and downstream alterations in cortical sensorimotor networks [3].

Our results are in line with this conceptualization: we observed both elevated TD thresholds and increased proprioceptive errors, which may represent complementary aspects of a more general sensory processing disorder in PD. However, the absence of significant correlations between these two domains suggests that, while both are impaired, they may arise from partially independent mechanisms. Several possible explanations can be considered. (i) Distinct neural substrates: TD is thought to depend primarily on basal ganglia–SMA interactions responsible for temporal encoding [8, 9, 33], whereas proprioceptive processing relies more on parietal and cerebellar integration of afferent inputs [34–36]. (ii) Modulatory influences of dopaminergic treatment: All patients were assessed in the clinically defined ON state. The observed dissociation between TD and proprioceptive measures may partly reflect medication effects, which could mask associations between domains. (iii) Task specificity and sensitivity: Although our paradigms were designed to isolate defined aspects of temporal and proprioceptive function, they cannot capture the full range of sensory impairments. The LED and ARROW tasks require active movement reproduction, engaging motor execution, attention, working memory, and visuospatial transformation (especially ARROW). These tasks primarily assess proprioceptive accuracy, but additional cognitive‐motor demands may contribute. The large ARROW error difference (PD 14.2° vs. HC 2.1°) likely reflects both factors; proprioception is probably the main contributor, though other processes cannot be excluded. More sensitive or multimodal assessments might reveal associations not detectable with the present approach.

In line with Conte et al. [3], sensory dysfunction in PD should not be viewed merely as a passive consequence of dopamine depletion but rather as reflecting fundamental disruptions in how sensory signals are filtered, weighted, and integrated within the central nervous system. From this perspective, the elevated TD thresholds and proprioceptive inaccuracies observed here may represent parallel manifestations of a broader dysfunction in sensory signal processing rather than expressions of a single underlying deficit. Interestingly, Lee et al. emphasize that TD abnormalities correlate more strongly with variability in motor performance than with global motor severity [32]. This may explain why no significant associations were found in our study between sensory measures and MDS‐UPDRS scores, which reflect averaged motor impairments rather than dynamic fluctuations or timing‐specific disturbances that may be more directly related to sensory dysfunction. The absence of correlations should therefore be interpreted as the absence of evidence in this cohort, not definitive evidence against associations.

### 4.5. Limitations

Several limitations should be acknowledged. First, the sample size was modest, limiting statistical power to detect subtle associations. Second, the inclusion of patients with DBS and heterogeneous treatment regimens introduced variability, although this reflects real‐world clinical populations. Third, all participants were assessed in the ON state, which restricts conclusions regarding medication‐ or stimulation‐dependent effects. Thus, the findings characterize treated PD in the ON condition and do not allow separation of disease‐related sensory dysfunction from acute dopaminergic effects. Fourth, despite clinical screening, subclinical peripheral sensory deficits cannot be fully excluded. Fifth, the experimental paradigms targeted specific aspects of TD and proprioception; incorporating additional modalities could provide a more comprehensive characterization of sensory function. Moreover, the proprioceptive tasks required active movement reproduction and therefore relied not only on afferent input but also on motor planning, attention, and working memory, which were not assessed separately. Finally, the cross‐sectional design precludes causal inferences and prevents evaluation of longitudinal changes.

### 4.6. Future Directions

Future studies should integrate behavioral, neurophysiological, and neuroimaging approaches to dissect the neural substrates of temporal and proprioceptive dysfunction. Longitudinal research is needed to track progression and treatment response. Stratification by clinical phenotype and intervention (e.g., DBS) may clarify variability in sensory deficits.

## 5. Conclusions

In conclusion, PD is associated with significant impairments in TD and proprioception, reflecting widespread sensory dysfunction beyond the classical motor syndrome. The lack of correlation between these domains suggests no evidence of association in this sample rather than definitive evidence for distinct pathophysiological mechanisms. These findings underscore the importance of considering sensory processing deficits as an integral component of PD and as potential targets for future therapeutic strategies.

## Author Contributions

T.M.O.: conceptualization, formal analysis, methodology, project administration, validation, and writingaoriginal draft preparation.

A.J.: formal analysis, investigation, visualization, and writing–original draft preparation.

J.V.: resources, supervision, validation, and writing–review and editing.

D.Z.: conceptualization, methodology, project administration, resources, supervision, and validation.

## Funding

This publication was supported by the Open Access Publication Fund of the University of Wuerzburg. Open Access funding enabled and organized by Projekt DEAL.

## Conflicts of Interest

The authors declare no conflicts of interest.

## Data Availability

The datasets generated during and/or analyzed during the current study are available from the corresponding author on reasonable request.
